# The Perinatal Risk Index: Early Risks Experienced by Domestic Adoptees in the United States

**DOI:** 10.1371/journal.pone.0150486

**Published:** 2016-03-24

**Authors:** Kristine Marceau, Marielena De Araujo-Greecher, Emily S. Miller, Suena H. Massey, Linda C. Mayes, Jody M. Ganiban, David Reiss, Daniel S. Shaw, Leslie D. Leve, Jenae M. Neiderhiser

**Affiliations:** 1 Rhode Island Hospital/Brown University, Providence, Rhode Island, United States of America; 2 The Pennsylvania State University, University Park, Pennsylvania, United States of America; 3 Northwestern University, Evanston, Illinois, United States of America; 4 Yale University, New Haven, Connecticut, United States of America; 5 George Washington University, Washington, D.C., United States of America; 6 University of Pittsburgh, Pittsburgh, Pennsylvania, United States of America; 7 University of Oregon, Eugene, Oregon, United States of America; Central South University, CHINA

## Abstract

We aimed to assess comprehensively the prevalence of perinatal risks experienced by a potentially high-risk yet understudied population of children domestically adopted in the United States. Data are from participant report and medical records from mothers (*n* = 580) who completed a domestic adoption placement with nonrelatives at or near birth (Mean placement age = 7 days). We describe a comprehensive measure of perinatal risks, including divergences from previous assessment tools and the incorporation of multiple reporters, and report the prevalence of various types of perinatal risks. The prevalence of each specific risk factor was generally low, although several risks were more prevalent in this sample than estimates from nationally representative publicly available data. Nearly the entire sample (99%) experienced some type of risk exposure. Birth mothers who placed their children for adoption domestically in the US experience higher levels of perinatal risks than the national average, but not for all specific types of risk. Thus, the developmental trajectories of children adopted domestically may systematically differ from the general population to the extent that these specific perinatal risks impact development.

## Introduction

A large and growing literature suggests that maternal experiences during pregnancy may impact child development [1–5]. Much of this literature relies on maternal self-report, which has been shown to be valid when collected appropriately [6]. Medical records and biomarker data are typically the gold-standards for assessing perinatal risks. In some cases, however, self-reports collected shortly after pregnancy may yield more complete information about perinatal experiences than medical record or biomarker data [7]. Maternal report may index a more global or pervasive problem potentially missed at specific obstetric visits, or may be more accurate if patients do not feel comfortable being entirely truthful with their obstetric provider (i.e., reporting substance use, psychiatric symptoms).

Our goal was to create a comprehensive measure to identify the wide variety of perinatal conditions that may pose risk to the fetus based on previous assessments [8], including more current guidelines [9], additional risks shown to affect child outcomes [10, 11], and multiple reporters. Based on the quality of the data from birth mother self-reports and medical records from prenatal visits and delivery, we created scores representing the most reliable, valid indicators of the various risks. We then examined the prevalence of the various types of risks in our putatively high-risk sample of mothers who completed domestic (United States) adoption plans whereby infants were placed at or near birth to nonrelatives. We report descriptive statistics on the variety and number of risks present and compare the prevalence of our most common perinatal risks with publically available data from the United States. Thus, this paper is the first to describe the perinatal risks experienced by this high-risk and under-studied sample in the literature, provides insights into the types of early life exposures domestically adopted children in the United States experience, and provides a comparison sample for other studies adopting a comprehensive approach to examining perinatal risks and child outcomes.

## Materials and Methods

### Participants and Procedures

Participants were drawn from a sample of 619 birth mothers with medical record and self-reported data on pregnancy and birth medical and psychiatric complications, recruited as a part of the Early Growth and Development Study (EGDS). EGDS is a multi-site prospective longitudinal adoption study tracking birth mothers and fathers, adoptive parents, and adopted children from birth through late childhood [12]. Mothers were eligible for participation if (1) the adoption was domestic, (2) the child was placed with a non-relative family (3) prior to 3 months of age (M = 7.11 days postpartum, SD = 13.28), (4) the child had no known major medical conditions, and (5) the birth mother (and adoptive parents) could read or understand English at the eighth-grade level. Recruitment occurred between March 2003 and January 2010, through 45 adoption agencies in 15 states. Adoption agencies contacted birth mothers who provided consent to be contacted by EGDS staff. Staff then called and explained the study and sent a consent form to birth mothers who were willing to participate. The full sample and procedures have been described in detail [12]. The research conducted here was approved by the Institutional Review Boards (IRBs) of all participating organizations (George Washington University, The Pennsylvania State University, University of California, Davis, University of Minnesota, Oregon Social Learning Center). Written informed consent was provided by the participants in this study at each assessment, as approved by the above IRBs. The current study used data from prenatal care, birth/delivery records, and interviews (usually in their homes) of birth mothers at ~4 months postpartum, and focuses on the perinatal experiences of the birth mothers. See Table 1 for sample demographics.

**Table 1:** Sample Descriptive Statistics.

	**Mean**	**SD**	**Range**
Maternal Age at child birth	24.57	6.17	13.71–45.10
**Race/Ethnicity**	**% of sample**		
Caucasian	70%		
Black or African American	13%		
Hispanic/Latino	7%		
More than one race/ethnicity	5%		
Other	5%		
**Marital Status**			
Single/Never married/Widowed	43%		
Living in committed relationship	32%		
Married	13%		
Separated	3%		
Divorced	9%		
**Education**			
Less than high school	20%		
High school or equivalent	53%		
Beyond high school	27%		
**Employment**			
Full-time employment	36%		
Part-time employment	15%		
Unemployed and looking for work	18%		
Full-time homemaker	8%		
Other	23%		
	**Mode**		**Range**
Modal personal income	>$15,000		>$15,000—$70,000–100,000
Modal household income	>$15,001		>$15,000—$200,001–300,000

Maternal age is presented in years. Beyond high school includes trade school, 2- and 4- year college degrees, and graduate study. Further sample details are provided in Leve et al. (2013).

### Measures

#### Self-report

We used two measures to assess perinatal risk via self-report. Interviewers helped mothers generate a list of life events (i.e., birthdays, holidays) that occurred around the pregnancy. These events were used to create a Life History Calendar [13, 14] relevant to the prenatal period (Pregnancy History Calendar) to aid women in recalling prenatal substance use (alcohol, cigarettes, illicit drugs) and symptoms of depression and anxiety during this time. Depression and anxiety symptoms were assessed on a scale of 0 (not at all) to 3 (severely) with seven items from the Beck Depression Inventory [15] and five items from the Beck Anxiety Inventory [16]. A subset of each original questionnaire was used to reduce burden on the mothers.

Mothers also completed a Pregnancy Screening questionnaire that assessed various medical aspects of the pregnancy, including timing and nature of recognition of pregnancy, weight changes, blood pressure, vitamin and prescription medication intake, laboratory tests, estimated due date, delivery date, timing and frequency of obstetric visits, and symptoms of illnesses (e.g., flu, sexually transmitted infections, pre-eclampsia).

#### Medical Records

We developed a coding form and manual (available upon author request) with close-ended questions assessing a diverse set of maternal and fetal complications, information on the completeness of prenatal care and birth/delivery records, mothers’ previous pregnancy outcomes, and other basic information. If the close-ended questions did not cover all of the information in the record, coders used open-ended questions to record the supplemental data exactly as it was presented in the medical records, writing as much as needed.

Reliability was achieved when 2+ coders had attained 100% agreement coding close-ended questions and 90% agreement coding open-ended questions on the same record. Reliability of each coder was re-checked every 10 records. If reliability was not met, the coder was instructed to code more practice records until reliability was achieved again. In addition to reliability checks prior to and during data collection, all records were triple coded to ensure all of the data were collected and recorded accurately. Some records were deemed illegible due to bad handwriting or very low copy quality, and were not used (*n* = 39 records, 6.3%); thus the analysis sample was 580 birth mothers.

#### Reporter differences

In cases where the specific risk factor was not present in one type of report, the other was used exclusively (e.g., we did not ask about diabetes in the self-reported data, but did code for it in the medical record data; therefore diabetes-related risk must be medical-record report). When medical records and self-report data assessed the same construct but were discordant, we used what we considered to be the most reliable, valid scores based on the following decisions:

Regarding the presence/absence of prenatal substance use, we utilized the source in which use was affirmatively reported, as both self-report and medical record data were expected to be similarly reliable (reports > 80% concordant across substances). The quantity and frequency of substance use recorded in medical records was typically vague and inadequate to determine the degree of intrauterine exposure. In contrast, the quantity and frequency of use of each of 12 addictive substances including alcohol and tobacco was assessed from participants, providing detail about the degree of use in the self-report data. Therefore, we always used self-reported data over medical record data for substance use severity which incorporated frequency and amount of use.

For risks related to pregnancy and birth complications (e.g., prematurity, postmaturity, low birth weight, perinatal infections, hypertension, pre-eclampsia), medical records data were preferable as information documented by obstetricians was deemed more accurate than that recalled by participants. For weight gain and weight loss during pregnancy, we used self-reported data if the birth mother had not seen an obstetric provider before 12 weeks of gestation. However, if she had begun prenatal care before 12 weeks, we used medical record report, as her pre-pregnancy weight would be more likely to be recorded accurately by the health care provider. For maternal age at birth, we used self-reported data because the maternal age at birth was calculated based on child and mother birth dates. Finally, for exposures to various toxins, we used the source that included affirmative report of exposure, as neither reporter was likely to be much more or less reliable than the other. Because medical records generally included only the presence or absence of psychiatric conditions, rather than specific symptoms, or the severity of these symptoms, participant reports of anxiety and depressive symptoms obtained using validated measures provided more robust indices of internalizing psychopathology experienced during pregnancy.

#### Perinatal Risk Index

We developed a comprehensive coding system for several classes of pregnancy risk (the Perinatal Risk Index) [17] based primarily on the McNeil-Sjöström obstetric complications scale (M-S) [8, 18]; and other resources (e.g., prenatal care visits [19], exposures to toxins [11]). Each risk factor was assigned a level of risk to the fetus (on a scale of 1–6) based on McNeil’s general obstetric and pediatric experiences, previous studies of pregnancy risk factors, and use of consultants [8]. This procedure was followed for all self-reported items and close-ended items in medical records. The content of the open-ended questions for each individual was screened by two PhD-level scientists and relevant risks not covered by the close-ended questions were aggregated and then assigned risk scores according to the M-S scale:

1. = Not harmful or relevant
2. = Not likely harmful or relevant
3. = Potentially but not clearly harmful or relevant
4. = Potentially clearly harmful or relevant
5. = Potentially clearly greatly harmful/relevant
6. = Very great harm to or deviation in offspring

Quartile scores identifying the rank of anxiety and depressive symptoms (independently) in the sample were used to index the severity of internalizing symptoms in the sample, in line with past research (e.g., “high” anxiety has been defined as the top 15% [20] or 25% [21] within a sample). We assigned the following risk scores in our sample to map onto the M-S scale: bottom 25% of the sample = 1, 25% to 50% = 2, 50%-75% = 3, 75%-85% = 4, and 85%-100% = 5.

Subscales: Eight indexes of pregnancy risk were created: one total score with five subscales, and two additional scales (described below). Each risk index was comprised of sums subtotals of items if applicable, and specific items when no subtotals are noted (see online supplement, S1 Table, for details):

1. Pregnancy Complications: maternal age-related risk (below age 18, 35–40, 40–45, >45 years), inadequate/late prenatal care, multiple gestation, intrauterine growth restriction, macrosomia, fetal heart rate deviations (e.g., fetal arrhythmia), fetal anemia, vaginal bleeding, placental abnormalities (e.g., single umbilical artery, placental previa), oligohydraminos, polyhydramnios, premature rupture of membranes, weight loss, weight gain outside of IOM guidelines, nausea, preeclampsia, kidney disease, chronic hypertension, maternal circulatory disorders (e.g., severe hypotension), noninfectious maternal respiratory disorders (e.g., asthma, bronchitis), maternal hormonal and metabolic disorders (e.g., diabetes, hypoglycemia, hypo/hyperthyroid), maternal gastrointestinal tract disorders (e.g., ulcerative colitis, appendicitis, diarrhea, dental infection), neurological conditions (e.g., epilepsy), maternal infections (e.g., urinary tract infections, upper respiratory infections, pyelonephrisis, otitis), maternal sexually transmitted infections (e.g., HIV, gonorrhea, chlamydia), other maternal trauma (e.g., abortion attempt, abdominal trauma, fractures).
2. Neonatal Complications: deviations in gestational age, birth weight, spina bifida, circulatory system malformations (e.g., pulmonary stenosis, heart murmur), genital tract malformations (e.g., hydronephrosis, cryptorchidism), craniosynostosis, fetal alcohol syndrome, congenital infections, hyperbilirubinemia, cephalohematoma, early neonatal complications (e.g., cyanosis, respiratory distress, low Apgar scores), meconium aspiration syndrome, atrial flutter, bradycardia, blood disorders (e.g., thrombocytopenia, polycythemia), endocrinological disorders (e.g., hypocalcemia, hypoglycemia), milia, sepsis, pneumonia, eye infections, hypothermia, and conditions requiring blood transfusion.
3. Maternal Substance Use: tobacco cigarettes, exposure to secondhand smoke (if not also smoking), alcohol, marijuana, cocaine, hallucinogens, amphetamines, heroin, prescription painkillers (used illegally), inhalants, sedatives, and tranquilizers.
4. Exposure to Toxins: exposure to radiation, lead, and chemical toxins.
5. Labor and Delivery: rupture of membranes, induced labor, labor length (prolonged labor or precipitous delivery), abnormal presentation, cephalopelvic disproportion, operative vaginal delivery (e.g., forceps or vacuum assisted vaginal delivery), cesarean delivery, intrapartum fetal heart rate tracing abnormalities (e.g., bradycardia, tachycardia, fetal distress), meconium stained amniotic fluid, umbilical cord complications during delivery (e.g., cord prolapse, cord compression, nuchal or body cord), placental abruption, postpartum hemorrhage, use of analgesics/anesthetics (e.g., epidural; general, spinal, opiate anesthesia).
6. Obstetric Complications Summary Score: Pregnancy Complications, Neonatal Complications, Substance Use, Exposure to Toxins, and Labor and Delivery Complications.
7. Previous Pregnancy Issues: terminations, miscarriages, previous preterm birth
8. Internalizing Symptoms: Anxiety and depression symptoms experienced during pregnancy

Summary scores: Total scores capturing the frequency of experiencing a risk that met the cutoff of potential risk to the fetus (e.g., a score of 3 or higher) were created to assess the number of risks present. To capture the severity of risk weighted risk totals [8, 18] are also available [17]. Here, we report the prevalence of risks using the total scores.

Departures from the McNeil-Sjöström scale: We made several departures from the M-S scale based on updated literature and current medical guidelines. In addition to the risks highlighted in the M-S scale, we also included maternal age at child birth as an additional risk in the pregnancy complications total score [10]. We included exposures to toxins (more toxins than listed in the original M-S scale) [11] and substance use during pregnancy in separate summary scores due to conceptual differences in these exposures from other pregnancy complications, and the growing literatures considering exposures to toxins and substance use during pregnancy specifically. Weight gain during pregnancy was updated to reflect changes in the Institute of Medicine guidelines using information on pre-pregnancy body mass index to judge adequate, inadequate, and excessive weight gain during pregnancy [9]. Finally, we added a previous pregnancy issues total score.

## Results

We present the overall statistics from our summary measures, as well as the most prevalent risks within each summary measure and a comparison from publicly available data when possible.

### Pregnancy Complications

On average, mothers experienced 2.30 pregnancy complications (at potentially but not clearly harmful or relevant levels or higher), SD = 1.40, Range = 0–8. Only 48 mothers (8.3%) experienced no pregnancy complications, whereas most mothers (*n* = 433, ~75.0%) experienced 1–3 distinct risks. Of the pregnancy complications, the top five most prevalent were sexually transmitted infections other than HIV (*n* = 317), high blood pressure (*n* = 276), excess weight gain (*n* = 77), maternal age-related risk (*n* = 142), and other infections (*n* = 123). The prevalence of high blood pressure in this sample was higher than a national estimate (47.6%, versus ~6–8% nationally) [22]. The prevalence of excess weight gain was lower than the national prevalence (24.9%, versus ~50% nationally) [23]. There were fewer very young and more somewhat older mothers than national estimates (under 19: *n* = 30, 5% versus ~14% nationally) [24] between 35 and 40: *n* = 148, 25% versus 10% nationally [25], between 40 and 45, *n* = 12, 2% versus 2% nationally [25], older than 45 years of age: *n* = 1. No comparison data was available for sexually transmitted infections other than HIV or other infections.

### Neonatal Complications

On average, mothers experienced 1.13 neonatal complications, SD = 0.71, Range = 0–7. Most mothers experienced no (*n* = 358, 61.7%), or only one neonatal complication (*n* = 179, 30.9%). The most prevalent were prematurity (*n* = 98), low Apgar score at 1, 5, or 10 minutes (*n* = 75), hyperbilirubinemia (*n* = 39), and low birth weight (*n* = 27). There were slightly more premature infants in our sample than nationally (16.9%, compared with 12–12.8% nationally) [26]. There were generally more low Apgar scores (which is associated with lower gestational age) than reported nationally (12.9%, versus 4% nationally) [27]. We had a similar prevalence of hyperbilirbinemia as a national estimate (6.7%, versus 3–6% nationally) [28]. Our rate of low birth weight was slightly lower than national prevalence (4.7%, versus 7.9–8.3% nationally) [26].

### Substance Use

On average, mothers used 1.13 substances, SD = 1.24, Range = 0–6. A large proportion of mothers did not use any substances (*n* = 234, 40.0%), although many used one (*n* = 165, 28.5%), or two or three (*n* = 153, 26.4%) substances. Cigarette use was the most prevalent (*n* = 233), followed by alcohol (*n* = 92), and marijuana (*n* = 89). There was some cocaine (*n* = 36), amphetamine (*n* = 51), sedative (*n* = 39), and prescription painkiller (*n* = 31) use. Heroin, hallucinogens, and inhalants were infrequently used (< 3.0% of the sample, combined). There was a higher rate of smoking during pregnancy, marijuana, and cocaine use during pregnancy in this sample than reported nationally (cigarettes: 40.0%, versus 20.4% nationally, marijuana: 15.3%, versus 2.9% nationally; cocaine: 6.2%, versus 1.1% nationally) [29, 30], but comparable rates of alcohol use (15.9%, versus 18.8% nationally) [29].

### Exposure to Toxins

On average, mothers were exposed to 0.38 types of toxins, SD = 0.53, Range = 0–2. The majority of mothers were not exposed to toxins (*n* = 374, 64.5%), or exposed to only one type (*n* = 193, 33.3%). Lead exposure was the most prevalent of the toxins (*n* = 189, 32.6%, no comparison data available).

### Labor and Delivery Complications

On average, mothers experienced 1.93 labor/delivery complications, SD = 1.37, Range = 0–7. Only 97 mothers (16.7%) experienced labor/delivery complications. The majority (*n* = 408, 70.0%) experienced 1–3 distinct labor/delivery complications. The most prevalent labor/delivery complications were induction of labor (*n* = 258), anesthesia (*n* = 344), cesarean delivery (*n* = 138), cord complications (*n* = 134), and intra-partum fetal heart rate tracing abnormalities (*n* = 105). There was more labor induction in this sample compared with that reported nationally (44.5%, versus ~23% nationally) [30]. The use of anesthesia was comparable to that reported nationally (59.3%, versus 61% nationally) [30]. The prevalence of C-section deliveries was slightly lower than the national estimates (23.8%, versus 33% nationally) [30]. We had higher prevalence of cord complications (23.1%, versus ~10% nationally) [31], but lower prevalence of intra-partum fetal heart rate tracing abnormalities (18.1%, versus ~39% nationally) [32].

### Obstetric Complications Total

The obstetric complications total represents the sum of all the specific risks cataloged. In total, on average mothers experienced 6.31 obstetric complications, SD = 2.43, range = 0–14. This was a fairly evenly distributed number of risks. Only one mother experienced no complications of any kind. 23.5% of the sample experienced four or fewer obstetric complications. Only 10.6% of the sample (*n* = 62) experienced 10 risks or more.

### Previous Pregnancy Issues

On average, mothers experienced 0.38 previous pregnancy risks, SD = 0.59, Range = 0–2. The majority of mothers had no previous pregnancy risks (*n* = 392, 67.6%), or experienced only one (*n* = 155, 26.7%). These were most often previous terminations (*n* = 135, 23.3%, no comparison data available), with some previous miscarriages (*n* = 86, 14.8%, versus ~11% nationally) [30].

### Internalizing Symptoms

30.2% of the sample was considered to reach risk levels of internalizing symptoms, M = 9.18, SD = 1.96 (20.0% for depression, M = 4.75, SD = 0.49 symptoms, 20.7% for anxiety, M = 5.84, SD = 0.37 symptoms). The top 50% of the sample experienced at least one symptom of anxiety or depression.

## Discussion

Virtually all pregnancies in our sample of mothers who placed their children for domestic adoption in the United States involved some type of perinatal risk, in contrast to previous reports from other types of samples. For example, in a case-control study, 80% of women without schizophrenia and 89% of schizophrenic participants experienced some risk [18]. More comprehensive measurement of potential perinatal risk captures more and more diverse experiences. Thus, we may expect this high level of overall risk because we assessed more risks than typically assessed, or because birth mothers may represent mothers who are at somewhat higher risk for pregnancy complications than the national average (as noted for some, but not all of the relatively prevalent specific risks here). This is certainly true for substance use during pregnancy, as 60% of the sample reported use of at least one substance (e.g., alcohol, cigarettes, marijuana or other illicit drugs).

Consistent with previous studies [18], the prevalence of most specific risks were low. Of the more prevalent risks, high blood pressure, maternal age between 35 and 40, prematurity, low Apgar, labor induction, cord complications, cigarette, marijuana and cocaine use were greater than national estimates. Thus, it would seem that our sample experienced higher perinatal risks than the national average, but not for all specific risks. On the whole, the children in this sample are healthy (see study inclusion criteria), although considered at somewhat higher risk of developing behavioral and other problems because of genetic and perinatal risk conveyed by the birth parents [33, 34]. Studies using this sample have found various perinatal risk factors to be associated with greater variability in hypothalamic-pituitary-adrenal axis functioning, lower executive functioning, and behavior problems in the children [17, 35–38]. Future studies are planned to examine specific mechanisms of perinatal risk for child development across domains, including behavioral and psychiatric symptoms and problems, endocrine development, academic achievement, and weight trajectories.

## Conclusion

We presented a novel and comprehensive measure of perinatal risk in a sample of mothers who placed a child for adoption domestically in the United States. More detailed information on the measure is available upon author request and in online supplements. We have characterized the types of perinatal risks experienced by an under-studied population in the literature. Using both medical records and self-reported data allowed for a more complete characterization of perinatal risks than either alone. However, even the self-report-only version of this measure has been shown to correlate with a wide variety of child outcomes [17, 35]. Consistent with other investigations, the prevalence of most specific risks are quite low; however, the accumulation of multiple risk factors is quite common and has important implications for child development. In the future, we believe this measure will be particularly useful for researchers investigating the role of the perinatal environment for child development.

## Supporting Information

S1 Table Constructs assessed in the Cohort I and II Self-report and Medical Record Report Versions of the Perinatal Index. Sections correspond to sections on the M-S scale. Risk range = possible risk scores in our data. X denotes that the risk was assessed in the self-report and/or medical record data. SR = self-report, MR = medical record.
(DOCX)
