# Supplementary material for: The Perinatal Risk Index: Early Risks Experienced by Domestic Adoptees in the United States
Source: PLoS One. 2016 Mar 24;11(3):e0150486. doi: 10.1371/journal.pone.0150486 (PMC4807102; doi:10.1371/journal.pone.0150486)
Supplement: S1 Table — Sections correspond to sections on the M-S scale. Risk range = possible risk scores in our data. X denotes that the risk was assessed in the self-report and/or medical record data. SR = self-report, MR = medical record. (DOCX) [file pone.0150486.s001.docx]

**S1 Table. Constructs assessed in the Cohort I and II Self-report and Medical Record Report Versions of the Perinatal Index**. Sections correspond to sections on the M-S scale. Risk range = possible risk scores in our data. X denotes that the risk was assessed in the self-report and/or medical record data. SR = self-report, MR = medical record.

| **Section** | **Risk Range** | **Variable** | **Self-report** | **Medical Records** | **Best Score** |
| --- | --- | --- | --- | --- | --- |
| **Pregnancy Complications** | | | | | |
| **PCA: Fetal/feto-placental conditions** | | | | | |
| **PCA.1 Multiple pregnancy** | | | | | |
|  | **3** | Multiple gestation |  | X | MR (only MR assessed) |
|  | **4** | Twin-to-twin transfusion syndrome (TTTS) |  | X | MR (only MR assessed) |
| **Subtotal** | | | | | |
|  | **3-4** | Multiple gestation subtotal |  | Maximum risk of the two preceding risks under PCA.1 | Maximum risk of the two preceding risks under PCA.1 |
| **PCA.2: Intrauterine growth restriction (IUGR)** | | | | | |
|  | **3** | Intrauterine growth restriction |  | X | MR (only MR assessed) |
| **PCA.3: Intrauterine growth acceleration** | | | | | |
|  | **3** | Macrosomia |  | X | MR (only MR assessed) |
| **PCA.4: Fetal heart rate deviations** | | | | | |
|  | **3** | Abnormal fetal heart rate tracing | (cohort 2 only) | X | MR (only MR assessed) |
|  | **6** | Absent fetal heartbeat | (cohort 2 only) | X | MR (only MR assessed) |
|  | **3** | Fetal heart rate abnormalities NOS |  | X | MR (only MR assessed) |
| **Subtotal** | | | | | |
|  | **3, 6** | Fetal heart rate abnormalities subtotal | Maximum risk of abnormal and absent fetal heart rate (cohort 2 only) | Maximum risk of the three preceding risks under PCA.4 | Maximum risk of the three preceding risks under PCA.4 |
| **PCA.5: Rh-immunization, ABO-immunization, fetal anemia** | | | | | |
|  | **4** | Fetal anemia |  | X | MR (only MR assessed) |
| **PCA.6: Diverse fetal deviations** | | | | | |
|  | **2** | Decreased fetal movement NOS | X | X | Maximum of SR and MR |
| **PCA.7: Bleeding from vagina during pregnancy** | | | | | |
|  | **4** | Bleeding NOS and bleeding late in pregnancy | (cohort 1 only) | X | MR (only MR assessed) |
|  | **5** | Placental abruption |  | X | MR (only MR assessed) |
|  | **3** | Spotting |  | X | MR (only MR assessed) |
|  | **5** | Early bleeding |  | X | MR (only MR assessed) |
|  | **4** | Antepartum hemorrhage |  | X | MR (only MR assessed) |
| **Subtotal** | | | | | |
|  | **3-5** | Bleeding from vagina subtotal |  | Maximum risk of the five preceding risks under PCA.7 | Maximum risk of the five preceding risks under PCA.7 |
| **PCA.8: Morphological and physio placetal(uterine) conditions** | | | | | |
|  | **4** | Single umbilical artery |  | X | MR (only MR assessed) |
|  | **3** | Placental previa |  | X | MR (only MR assessed) |
|  | **2** | Placenta calcification |  | X | MR (only MR assessed) |
|  | **2** | Placental infarction |  | X | MR (only MR assessed) |
| **Subtotal** | | | | | |
|  | **3-4** | Placenta conditions subtotal |  | Maximum risk of One artery in umbilical cord and Placental previa | Maximum risk of One artery in umbilical cord and Placental previa |
|  | **2** | Placenta degeneration subtotal |  | Maximum risk of Calcium Deposit on Placenta and Placental Infarcts | Maximum risk of Calcium Deposit on Placenta and Placental Infarcts |
| **PCA.10: Other conditions** | | | | | |
|  | **4** | Oligohydraminos |  | X | MR (only MR assessed) |
|  | **3** | Polyhydramnios |  | X | MR (only MR assessed) |
|  | **1** | Braxton hicks contractions |  | X | MR (only MR assessed) |
|  | **5** | Premature rupture of membrane |  | X | MR (only MR assessed) |
|  | **2** | Cervical insufficiency |  | X | MR (only MR assessed) |
| **Subtotal** | | | | | |
|  | **1-5** | Other conditions subtotal |  | Maximum risk of the five preceding scores under PCA.10 | Maximum risk of the five preceding scores under PCA.10 |
| **PCB** | | | | | |
| **PCB.1 Maternal Disorders: includes kidney disease/albuminuria, (pre)eclampsia, hypertension, weight gain/edema** | | | | | |
|  | **3** | Hypertension | X | X | MR (likely to be more accurate) |
|  | **4** | Preeclampsia | (Cohort 2 only) | X | MR (likely to be more accurate) |
|  | **4** | Kidney disease |  | X | MR (only MR assessed) |
|  | **2-3** | Albuminuria |  | X | MR (only MR assessed) |
|  | **2** | Proteinuria |  | X | MR (only MR assessed) |
|  | **2-3** | Excessive weight gain | X | X | MR if prenatal care before 1^st^ trimester. SR if limited prenatal care |
|  | **2** | Obesity | X | X | Maximum of MR and SR |
|  | **1** | Edema | (cohort 1 only) | X | MR (only MR assessed) |
|  | **5** | HELLP syndrome |  | X | MR (only MR assessed) |
| **Subtotal** | | | | | |
|  | **1-5** | Maternal disorders subtotal | Maximum risk of Obesity, weight gain, blood pressure | Maximum risk of the nine preceding scores under PCB.1 | Maximum risk of the nine preceding scores under PCB.1 |
| **PCB.2: other maternal noninfectious urinary tract disorders** | | | | | |
|  | **2** | Nephrolithiasis |  | X | MR (only MR assessed) |
|  | **2** | Hematuria |  | X | MR (only MR assessed) |
|  | **1** | Clinically significant hydronephrosis |  | X | MR (only MR assessed) |
| **Subtotal** | | | | | |
|  | **1-2** | Noninfectious urinary tract disorders subtotal |  | Maximum risk of the three preceding scores under PCB.2 | Maximum risk of the three preceding scores under PCB.2 |
| **PCB.3: low maternal weight gain, hyperemesis and poor nutrition** | | | | | |
|  | **3-4** | Loss of appetite/weight loss | X | X | MR if prenatal care before 1^st^ trimester. SR if limited prenatal care |
|  | **3** | Underweight NOS |  | X | MR (only MR assessed) |
|  | **4** | Hyperemesis gravidarum |  | X | MR (only MR assessed) |
| **Subtotal** | | | | | |
|  | **3-4** | Low weight gain/poor nutrition subtotal |  | Maximum risk of the three preceding scores under PCB.3 | Maximum risk of the three preceding scores under PCB.3 |
| **PCB.4: other maternal circulatory disorders** | | | | | |
|  | **2** | Anemia [mother] | (cohort 2 only) | X | MR (only MR assessed) |
|  | **2** | Heart disease [mother] |  | X | MR (only MR assessed) |
|  | **2** | Arrhythmia [mother] |  | X | MR (only MR assessed) |
|  | **1** | Heart murmur [mother] |  | X | MR (only MR assessed) |
|  | **3** | Severe hypotension [medically confirmed] |  | X | MR (only MR assessed) |
| **Subtotal** | | | | | |
|  | **1-3** | Maternal circulatory disorders subtotal |  | Maximum risk of the five preceding scores under PCB.4 | Maximum risk of the five preceding scores under PCB.4 |
| **PCB.5: noninfectious maternal respiratory disorders** | | | | | |
|  | **3** | Asthma [mother] |  | X | MR (only MR assessed) |
|  | **3** | Bronchitis [mother] |  | X | MR (only MR assessed) |
| **Subtotal** | | | | | |
|  | **3** | Noninfectious maternal respiratory disorders subtotal |  | Maximum risk of the two preceding scores under PCB.5 | Maximum risk of the two preceding scores under PCB.5 |
| **PCB.6: maternal hormonal and metabolic disorders** | | | | | |
|  | **4** | Diabetes | (cohort 2 only) | X | MR (only MR assessed) |
|  | **2** | Glycosuria NOS |  | X | MR (only MR assessed) |
|  | **4** | Hypoglycemia |  | X | MR (only MR assessed) |
|  | **3** | Hypothyroid NOS |  | X | MR (only MR assessed) |
|  | **4** | Hyperthyroid NOS |  | X | MR (only MR assessed) |
| **Subtotal** | | | | | |
|  | **3-4** | Hormonal disorders subtotal |  | Maximum risk of hypothyroid NOS and hyperthyroid NOS | Maximum risk of hypothyroid NOS and hyperthyroid NOS |
|  | **2, 4** | Diabetes-related risk subtotal |  | Maximum risk of diabetes, glycosuria NOS, and hypoglycemia | Maximum risk of diabetes, glycosuria NOS, and hypoglycemia |
| **PCB.7 maternal gastrointestinal tract disorders** | | | | | |
|  | **2** | Gastritis |  | X | MR (only MR assessed) |
|  | **2** | Epigastric pain |  | X | MR (only MR assessed) |
|  | **4** | Ulcerative colitis |  | X | MR (only MR assessed) |
|  | **2** | Cholelithiasis |  | X | MR (only MR assessed) |
|  | **2-3** | Diarrhea |  | X | MR (only MR assessed) |
|  | **3** | Dental infection |  | X | MR (only MR assessed) |
|  | **4** | Appendicitis |  | X | MR (only MR assessed) |
| **Subtotal** | | | | | |
|  | **2-4** | Gastrointestinal tract disorders subtotal |  | Maximum risk of the seven preceding scores under PCB.7 | Maximum risk of the seven preceding scores under PCB.7 |
| **PCB.8: neurological conditions common type** | | | | | |
|  | **4-5** | Epilepsy | (cohort 2 only) | X | MR (only MR assessed) |
|  | **2** | Migraine |  | X | MR (only MR assessed) |
| **Subtotal** | | | | | |
|  | **2-5** | Neurological conditions subtotal |  | Maximum risk of the two preceding scores under PCB.8 | Maximum risk of the two preceding scores under PCB.8 |
| **PCB.11: Maternal Infections** | | | | | |
|  | **2-4** | Urinary infection | X | X | MR (likely to be more accurate) |
|  | **3-5** | Rubella | X | X | MR (likely to be more accurate) |
|  | **2** | Influenza | X | X | MR (likely to be more accurate) |
|  | **3** | Upper respiratory infection | X | X | MR (likely to be more accurate) |
|  | **3** | Fever/Chills | (cohort 1 only) | X | MR (only MR assessed) |
|  | **3** | Cold |  | X | MR (only MR assessed) |
|  | **2-4** | Pyelonephritis |  | X | MR (only MR assessed) |
|  | **2** | Cystitis |  | X | MR (only MR assessed) |
|  | **3** | Tonsillitis |  | X | MR (only MR assessed) |
|  | **3** | Sinusitis |  | X | MR (only MR assessed) |
|  | **3** | Otitis |  | X | MR (only MR assessed) |
|  | **2** | Virual infection NOS |  | X | MR (only MR assessed) |
|  | **2** | Skin infections |  | X | MR (only MR assessed) |
|  | **3** | Yeast infection |  | X | MR (only MR assessed) |
|  | **4** | Pneumonia |  | X | MR (only MR assessed) |
|  | **4** | Toxoplasma |  | X | MR (only MR assessed) |
|  | **3** | Hepatitis C |  | X | MR (only MR assessed) |
|  | **5** | Erythema |  | X | MR (only MR assessed) |
|  | **6** | HIV/AIDS | X | X | MR (only MR assessed) |
|  |  | STDs NOS | (cohort 2 only) |  | n/a for consistency across cohorts |
|  | **4** | Gonorrhea |  | X | MR (only MR assessed) |
|  | **3** | Chlamydia |  | X | MR (only MR assessed) |
|  | **4** | Syphilis |  | X | MR (only MR assessed) |
|  | **3** | HPV |  | X | MR (only MR assessed) |
|  | **3** | Abnormal Pap |  | X | MR (only MR assessed) |
|  | **3** | Herpes |  | X | MR (only MR assessed) |
|  | **3** | Genital Warts |  | X | MR (only MR assessed) |
|  | **3** | Bacterial Vaginosis |  | X | MR (only MR assessed) |
|  | **3** | Vaginosis candida |  | X | MR (only MR assessed) |
|  | **3** | GBS+ |  | X | MR (only MR assessed) |
|  | **3** | Other STDS |  | X | MR (only MR assessed) |
| **Subtotals** | | | | | |
|  | **2-5** | Maternal infections subtotal | Maximum of the scores for urinary tract infection, rubella, flu, upper respiratory infection | Maximum of the preceding scores from urinary tract infections through erythema | Maximum of the preceding scores from urinary tract infections through erythema |
|  | **3-6** | Maternal sexually-transmitted infections subtotal | Maximum of STDs NOS and HIV/AIDS | Maximum of the preceding scores from HIV/AIDS through other STDS | Maximum of the preceding scores from HIV/AIDS through other STDS |
| **PCB.12: minor disorders of pregnancy** | | | | | |
|  | **2** | Severe Nausea, Vomiting, and/or abdominal pain | X | X | n/a (can’t reach risk cutoff) |
|  | **1** | Heartburn |  | X | MR (only MR assessed) |
|  | **1** | fatigue only during pregnancy |  | X | MR (only MR assessed) |
|  | **2** | Headache |  | X | MR (only MR assessed) |
|  | **2** | vertigo |  | X | MR (only MR assessed) |
|  | **1** | Varicose veins |  | X | MR (only MR assessed) |
|  | **3** | Fainting (orthostatic) |  | X | MR (only MR assessed) |
|  | **2** | Backpain |  | X | MR (only MR assessed) |
|  | **2** | Pelvic pain |  | X | MR (only MR assessed) |
|  | **1** | Constipation |  | X | MR (only MR assessed) |
|  | **2** | Allergies |  | X | MR (only MR assessed) |
|  | **1** | Hemmorrhoids |  | X | MR (only MR assessed) |
|  | **2** | Tooth extraction |  | X | MR (only MR assessed) |
| **PCC: Medical examinations and interventions** | | | | | |
|  | **3** | Amniocentesis | (cohort 2 only) |  | n/a for consistency across cohorts |
| **PCD: Maternal Toxins/Radiation** | | | | | |
|  | **2** | X-Rays | X | X | Maximum score of SR and MR |
|  | **3** | Exposure to lead | X | X | Maximum score of SR and MR |
|  | **3** | Chemical toxins | X | X | Maximum score of SR and MR |
|  | **3** | Radiation | X |  | SR (only SR assessed) |
| **PCF: Maternal Street Drugs *Severity*** | | | | | |
|  | **3** | Cigarettes | X | X | SR (MR data of poor quality) |
|  | **3** | Second-hand smoke | X | X | SR (MR data of poor quality) |
|  | **3** | Alcohol | X | X | SR (MR data of poor quality) |
|  | **2-4** | Marijuana | X | X | SR (MR data of poor quality) |
|  | **4-6** | Cocaine/crack | X | X | SR (MR data of poor quality) |
|  | **3-6** | Hallucinogens | X | X | SR (MR data of poor quality) |
|  | **2-4** | Inhalants | X | X | SR (MR data of poor quality) |
|  | **4-6** | Methamphetamine | X | X | SR (MR data of poor quality) |
|  | **4-6** | Heroin | X | X | SR (MR data of poor quality) |
|  | **4-6** | Prescription pain killers | X | X | SR (MR data of poor quality) |
|  | **4-6** | Sedatives (opiates) | X | X | SR (MR data of poor quality) |
|  | **4-6** | Tranquilizers | X |  | SR (only SR assessed) |
|  | **5-6** | Methadone | (cohort 2 only) | X | SR (MR data of poor quality) |
|  | **4-6** | Other drug use NOS |  | X | Not included questionable quality |
|  | **3-4** | Caffeine |  | X | Not included questionable quality |
| **PCG: Other Maternal Trauma** | | | | | |
|  | **5** | Abortion attempt |  | X | MR (only MR assessed) |
|  | **4** | Abdominal trauma |  | X | MR (only MR assessed) |
|  | **2** | Fracture |  | X | MR (only MR assessed) |
| **Labor and Delivery Complications** | | | | | |
| **LDC Section B. Timing and rupture of placental membranes** | | | | | |
|  | **3-4** | Rupture of membranes |  | X | MR (only MR assessed) |
| **LDC Section C. Induction and stimulation of labor** | | | | | |
|  | **2-3** | Induction (labor) | (cohort 2 only) | X | MR (only MR assessed) |
| **LDC section D. Length of labor** | | | | | |
|  | **4** | Prolonged labor | (cohort 2 only) | X | MR (only MR assessed) |
|  | **3** | Tocolysis for Inertia of labor NOS |  | X | MR (only MR assessed) |
|  | **3-4** | Precipitous labor |  | X | MR (only MR assessed) |
| **Subtotal** | | | | | |
|  | **3-4** | Labor length subtotal |  | Maximum of the three preceding scores under LDC.D | Maximum of the three preceding scores under LDC.D |
| **LDC section G. Fetal presentation at delivery** | | | | | |
|  | **4** | Abnormal presentation | (cohort 2 only) | X | MR (only MR assessed) |
| **LDC section I. Pelvic abnormality feto-pelvic disproportionality, uterine abnormality** | | | | | |
|  | **4** | Placenta previa without bleeding |  | X | MR (only MR assessed) |
|  | **4** | Cephalopelvic distortion |  | X | MR (only MR assessed) |
| **Subtotal** | | | | | |
|  | **4** | Pelvic abnormality subtotal |  | Maximum of the two preceding scores under LDC.I | Maximum of the two preceding scores under LDC.I |
| **LDC section J. Operative delivery or intervention** | | | | | |
|  | **4** | Cesearean delivery | (cohort 2 only) | X | MR (only MR assessed) |
|  | **4** | Forceps assisted delivery |  | X | MR (only MR assessed) |
|  | **3** | Vacuum assisted delivery |  | X | MR (only MR assessed) |
| **LDC section K. Intrapartum fetal asphyxia conditions** | | | | | |
|  | **4** | Bradycardia (during delivery) NOS |  | X | MR (only MR assessed) |
|  | **3** | Tachycardia (during delivery) NOS |  | X | MR (only MR assessed) |
|  | **5** | Fetal distress NOS |  | X | MR (only MR assessed) |
|  | **5** | Both brady + tachycardia |  | X | MR (only MR assessed) |
|  | **4** | Meconium in amniotic fluid |  | X | MR (only MR assessed) |
|  | | | | | |
|  | **3-5** | Intrapartum fetal heart rate abnormalities |  | Maximum of the five preceding scores under LDC.K | Maximum of the five preceding scores under LDC.K |
| **LDC section L. Cord complications during delivery** | | | | | |
|  | **6** | Cord prolapse | (cohort 2 only) | X | MR (only MR assessed) |
|  | **3** | Cord compression |  | X | MR (only MR assessed) |
|  | **3-4** | Body/nuchal cord |  | X | MR (only MR assessed) |
| **Subtotal** | | | | | |
|  | **3-6** | Cord complications subtotal |  | Maximum of the three preceding scores under LDC.L | Maximum of the three preceding scores under LDC.L |
| **LDC section N. Bleeding during labor** | | | | | |
|  | **4** | Postpartum hemorrhage |  | X | MR (only MR assessed) |
|  | **5** | Placental abruption |  | X | MR (only MR assessed) |
| **Subtotal** | | | | | |
|  | **4-5** | Bleeding during labor subtotal |  | Maximum of the two preceding scores under LDC.N | Maximum of the two preceding scores under LDC.N |
| **LDC section P. Analgesics, Anesthetics, & Pharmacology** | | | | | |
|  | **3** | Epidural | (cohort 2 only) | X | MR (only MR assessed) |
|  | **4** | Narcotics during delivery | (cohort 2 only) |  | n/a not measured on everyone |
|  | **3** | Sedatives during delivery | (cohort 2 only) |  | n/a not measured on everyone |
|  | **4** | General Anesthesia NOS |  | X | MR (only MR assessed) |
|  | **4** | Spinal anesthesia |  | X | MR (only MR assessed) |
|  | **2** | Local anesthesia NOS |  | X | MR (only MR assessed) |
|  | **4** | Opiate anesthesia |  | X | MR (only MR assessed) |
| **Subtotal** | | | | | |
|  | **2-4** | Anesthetics subtotal | Maximum risk of epidural, narcotics during delivery, and sedatives during delivery | Maximum risk of epidural, general, local, spinal, and opiate anesthesia | Maximum risk of epidural, general, local, spinal, and opiate anesthesia |
| **Neonatal Complications** | | | | | |
| **NC A: Deviations in gestational age, weight, and maturation at birth** | | | | | |
|  | **4-6** | Prematurity | X | X | MR (likely to be more accurate) |
|  | **4** | Postmaturity | X | X | MR (likely to be more accurate) |
|  | **4** | Low Birthweight | X | X | MR (likely to be more accurate) |
| **Subtotal** | | | | | |
|  | **4-6** | Deviations in gestational age, weight and maturation subtotal | Maximum risk of the three preceding scores under NC.A | Maximum risk of the three preceding scores under NC.A | Maximum risk of the three preceding scores under NC.A |
| **NC B: Congenital structural malformations** | | | | | |
| **NC.B1: CNS-related malformations** | | | | | |
|  | **4** | Spina bifida |  | X | MR (only MR assessed) |
| **NC.B3: Circulatory system malformations** | | | | | |
|  | **4** | Pulmonary stenosis |  | X | MR (only MR assessed) |
|  | **3** | Heart murmur NOS @ delivery |  | X | MR (only MR assessed) |
|  | **4** | Patent Ductus Arteriosus |  | X | MR (only MR assessed) |
| **Subtotal** | | | | | |
|  | **3-4** | Circulatory system malfunctions subtotal |  | Maximum risk of the three preceding scores under NC.B3 | Maximum risk of the three preceding scores under NC.B3 |
| **NC.B9: Genital tract malformations** | | | | | |
|  | **3** | Hydronephrosis |  | X | MR (only MR assessed) |
|  | **3** | Cryptorchidism |  | X | MR (only MR assessed) |
| **Subtotal** | | | | | |
|  | **3** | Genital tract malfunctions subtotal |  | Maximum risk of the two preceding scores under NC.B9 | Maximum risk of the two preceding scores under NC.B9 |
| **NC.B10: Musculoskeletal malformations** | | | | | |
|  | **4** | Craniosynostosis |  | X | MR (only MR assessed) |
| **NC.B13: General malformation syndromes** | | | | | |
|  | **6** | Fetal Alcohol Syndrome |  | X | MR (only MR assessed) |
| **NC D: Congenital infections** | | | | | |
|  | **5** | Congenital Infections |  | X | MR (only MR assessed) |
| **NC E: Neonatal disorders** | | | | | |
| **NCE.1 block 1: nervous system, brain, head, and skull** | | | | | |
|  | **4** | Hyperbilirubinemia |  | X | MR (only MR assessed) |
|  | **5** | Withdrawal symptoms (from maternal drug use) |  | X | MR (only MR assessed) |
| **Subtotal** | | | | | |
|  | **4-5** | Neonatal CNS disorders subtotal |  | Maximum of the preceding two scores under NCE.1 block 1 | Maximum of the preceding two scores under NCE.1 block 1 |
| **NCE.1 block 3: cerebral hemorrahage** | | | | | |
|  | **4** | Cephalohematoma NOS |  | X | MR (only MR assessed) |
| **NCE.2: Early period general condition** | | | | | |
|  | **4** | Cyanosis NOS |  | X | MR (only MR assessed) |
|  | **4** | Required resuscitation after birth |  | X | MR (only MR assessed) |
|  | **4** | Apnea |  | X | MR (only MR assessed) |
|  | **4** | Respiratory distress NOS |  | X | MR (only MR assessed) |
|  | **3** | Tachypnea NOS |  | X | MR (only MR assessed) |
|  | **4** | Required oxygen support |  | X | MR (only MR assessed) |
|  | **4-6** | Low Apgar score | (cohort 2 only, yes/no) | Calculated from Apgar scores at 1, 5, and 10 minutes | MR (likely to be more accurate) |
| **Subtotal** | | | | | |
|  | **3-6** | Early general conditions subtotal |  | Maximum of the seven preceding scores under NCE.2 | Maximum of the seven preceding scores under NCE.2 |
| **NCE.3 block 2: lung disorders** | | | | | |
|  | **5** | Meconium aspiration syndrome |  | X | MR (only MR assessed) |
| **NCE.3 block 3: arrhythmia** | | | | | |
|  | **4** | Atrial Flutter |  | X | MR (only MR assessed) |
|  | **5** | Bradycardia |  | X | MR (only MR assessed) |
| **Subtotal** | | | | | |
|  | **4-5** | Arrhythmia subtotal |  | Maximum of the two preceding scores under NCE.3 | Maximum of the two preceding scores under NCE.3 |
| **NC.E9: Blood disorders** | | | | | |
|  | **5** | Thrombocytopenia |  | X | MR (only MR assessed) |
|  | **3** | Vitamin K deficiency |  | X | MR (only MR assessed) |
|  | **5** | Polycythemia |  | X | MR (only MR assessed) |
| **Subtotal** | | | | | |
|  | **3-5** | Blood disorders subtotal |  | Maximum of the three preceding scores under NC.E9 | Maximum of the three preceding scores under NC.E9 |
| **NCE.10: Endocrinological disorders** | | | | | |
|  | **4** | Hypocalcemia |  | X | MR (only MR assessed) |
|  | **4** | Hypoglycemia |  | X | MR (only MR assessed) |
| **NCE.12: Skin disorders** | | | | | |
|  | **2** | Milia |  | X | MR (only MR assessed) |
| **NC.E13: New neonatal infections** | | | | | |
|  | **6** | Sepsis |  | X | MR (only MR assessed) |
|  | **5** | Pneumonia |  | X | MR (only MR assessed) |
|  | **3** | Eye infection |  | X | MR (only MR assessed) |
| **NCE.14: General disorders, signs and symptoms** | | | | | |
|  | **4** | Low body temperature NOS |  | X | MR (only MR assessed) |
| **NCF: Treatment, including offspring medications** | | | | | |
|  | **4** | Blood transfusion |  | X | MR (only MR assessed) |
| **Previous Pregnancies** | | | | | |
|  | **3** | Terminations | (cohort 2 only) | X | MR (only MR assessed) |
|  | **3** | Miscarriages | (cohort 2 only) | X | MR (only MR assessed) |
|  | **3** | Stillbirths | (cohort 2 only) |  | SR (only SR assessed) |
|  | **2** | Previous preterm births |  | X | MR (only MR assessed) |
| **Maternal Psychiatric Problems during Pregnancy** | | | | | |
|  | **3-5** | Depression | Based on symptom thresholds | Presence/absence of disorder | SR (MR of poor quality) |
|  | **3-5** | Anxiety | Based on symptom thresholds | Presence/absence of disorder | SR (MR of poor quality) |
| **Other Variables** | | | | | |
|  | **3-5** | Maternal age (risk score) | X | X | SR: calculated from maternal date of birth and delivery date |
|  | **1-5** | Prenatal care | X |  | SR (only SR collected) |
